# Effect of nitrogen application on enhancing high-temperature stress tolerance of tomato plants during the flowering and fruiting stage

**DOI:** 10.3389/fpls.2023.1172078

**Published:** 2023-06-08

**Authors:** Jing Luo, Zaiqiang Yang, Fengyin Zhang, Chunying Li

**Affiliations:** Jiangsu Key Laboratory of Agricultural Meteorology, School of Applied Meteorology, Nanjing University of Information Science and Technology, Nanjing, China

**Keywords:** high-temperature stress, nitrogen, tomato, abiotic stress tolerance, principal component analysis, comprehensive evaluation

## Abstract

This study was conducted to investigate the effects of nitrogen application on growth, photosynthetic performance, nitrogen metabolism activities, and fruit quality of tomato plants under high-temperature (HT) stress. Three levels of daily minimum/daily maximum temperature were adopted during the flowering and fruiting stage, namely control (CK; 18°C/28°C), sub-high temperature (SHT; 25°C/35°C), and high-temperature (HT; 30°C/40°C) stress. The levels of nitrogen (urea, 46% N) were set as 0 (N_1_), 125 (N_2_), 187.5 (N_3_), 250 (N_4_), and 312.5 (N_5_) kg hm^2^, respectively, and the duration lasted for 5 days (short-term). HT stress inhibited the growth, yield, and fruit quality of tomato plants. Interestingly, short-term SHT stress improved growth and yield via higher photosynthetic efficiency and nitrogen metabolism whereas fruit quality was reduced. Appropriate nitrogen application can enhance the high-temperature stress tolerance of tomato plants. The maximum net photosynthetic rate (*P*_Nmax_), stomatal conductance (*g*_s_), stomatal limit value (L_S_), water-use efficiency (WUE), nitrate reductase (NR), glutamine synthetase (GS), soluble protein, and free amino acids were the highest in N_3_, N_3_, and N_2_, respectively, for CK, SHT, and HT stress, whereas carbon dioxide concentration (*C*_i_), was the lowest. In addition, maximum SPAD value, plant morphology, yield, Vitamin C, soluble sugar, lycopene, and soluble solids occurred at N_3_-N_4_, N_3_-N_4_, and N_2_-N_3_, respectively, for CK, SHT, and HT stress. Based on the principal component analysis and comprehensive evaluation, we found that the optimum nitrogen application for tomato growth, yield, and fruit quality was 230.23 kg hm^2^ (N_3_-N_4_), 230.02 kg hm^2^ (N_3_-N_4_), and 115.32 kg hm^2^ (N_2_), respectively, at CK, SHT, and HT stress. Results revealed that the high yield and good fruit quality of tomato plants at high temperatures can be maintained by higher photosynthesis, nitrogen efficiency, and nutrients with moderate nitrogen.

## 1 Introduction

Tomato (*Solanum lycopersicum* L.) is one of the most widely grown, consumed, and produced crops in the greenhouse worldwide (Liu and Wang, 2020). It has attracted attention due to its rich Vitamin C and other nutrients, especially lycopene, which is related to its effects as a natural antioxidant (Alenazi et al., 2020; Imran et al., 2020).

Global warming has continued to raise concerns in recent years (Marlon et al., 2021; Hoffmann et al., 2022). The effect of global warming may cause irreversible damage to the growth and development of plants (Mathur et al., 2014; Ezquer et al., 2020). Tomato is a temperature-loving crop and is sensitive to temperature throughout fertility (Birje et al., 2020; Hao et al., 2020). The optimum growth temperature of tomatoes is between 18°C and 32°C (Berry and Bjorkman, 1980; Van Ploeg and Heuvelink, 2005). Prolonged exposure to temperature exceeding this range will restrict the growth and production of tomatoes more frequently and seriously (Bertin et al., 2000; Nicola et al., 2009). Therefore, global warming will negatively affect the growth and productivity of tomato plants, especially in the summer seasons.

High temperature (HT) has adverse effects on photosynthesis, such as significantly reducing the maximum net photosynthetic rate (*P*_Nmax_), apparent quantum efficiency (AQE), and light-compensation point (LCP), and significantly increasing the light-saturation point (LSP) (Djanaguiraman et al., 2020; Li et al., 2023). Previous studies demonstrated that gas exchange and photosynthesis had marked reduction under HT stress due to the inhibition of chloroplast and photosystem II (PSII) activity (Salvucci and Crafts-Brandner, 2004; Allakhverdiev et al., 2008). HT stress also reduces nitrogen metabolism via lower photosynthesis, resulting in the loss of nutrients (Prasad et al., 2008; Hammer et al., 2018). In addition, HT stress also influences morphological symptoms in tomato plants after the decline of photosynthesis and metabolism appear. It is well known that HT stress can reduce cell division and restrict cell elongation, thereby delaying plant growth (Ashraf and Harris, 2004; Camejo et al., 2005). Since plants cannot maintain some fundamental processes, HT stress may severely affect plant growth, yield, and fruit quality (Wahid et al., 2007; Bita and Gerats, 2013).

Although high temperatures have many adverse effects on plants, nitrogen application can enhance high-temperature stress tolerance (James et al., 2018). First, nitrogen can increase the photosynthesis and the scavenging capacity of reactive oxygen species, which in turn improves leaves to resist high-temperature adversity (Liu et al., 2019). Second, the smooth and efficient functioning of the nitrogen metabolic system in the leaves is ensured with increased nitrogen application, improving the nitrogen metabolism, and reducing the damage caused by HT stress (Iqbal et al., 2020; Ru et al., 2022). Specifically, excessive nitrogen application at high-temperature stress will exacerbate the damage to plants, which is characterized by lower yield (Peng et al., 2017).

Plants will continue to be challenged by HT stress in the future. Temperature and nitrogen both play important roles in plant growth (Sun et al., 2012; Alvar-Beltrán et al., 2019). However, to our knowledge, little is known about the effects of nitrogen application on tomato growth, development, and fruit quality under HT stress. In our study, we hypothesize that appropriate nitrogen would effectively increase the photosynthetic progress, and nitrogen metabolism and further affect the yield and fruit quality of tomato plants. Our objectives were (1) to compare the growth, photosynthetic performance, nitrogen metabolism activities, and fruit quality of tomatoes under different temperatures, (2) to investigate the effects of nitrogen application on the growth, yield, and fruit quality of tomatoes under high-temperature stress, (3) to determine the optimum nitrogen level to be applied under different high-temperature conditions, hoping to provide scientific contribution for high yield and good fruit quality of tomatoes in the greenhouse.

## 2 Materials and methods

### 2.1 Plant material and experimental treatments

The experiments were conducted in a Venlo-type greenhouse of the Nanjing University of Information Science and Technology (NUIST) from March 2022 to August 2022. Tomato seedlings (*Solanum lycopersicum* L., “Caesar”) were planted into pots of 30 cm (height) × 30 cm (upper diameter) × 25 cm (lower diameter) filled with peat soil: perlite: vermiculite = 2:1:1 (v/v/v). The soil was a medium loam with pH=7.4, 12.93 g kg^-1^organic carbon (C), 22.29 g kg^-1^ organic matter content, 69.36 mg kg^-1^ effective phosphorus (P), 13.80 mg L^-1^ available potassium (K), and 0.13% total nitrogen (N). To ensure proper soil nutrient content, base fertilizers were applied in phosphorus (calcium superphosphate, 12%P_2_O_5_, 200 kg hm^2^) and potassium (potassium sulfate, 52%K_2_O, 300 kg hm^2^). Five levels of nitrogen (urea, 46%N) were set as 0 (0N, N_1_), 125 (0.5N, N_2_), 187.5 (0.75N, N_3_), 250 (1.0N, N_4_), and 312.5 (1.25N, N_5_) kg hm^2^ (250 kg hm^2^ as the percentage of standard nutrient requirements (Yang et al., 2017; Wang et al., 2020)). The ratio of nitrogen was 30%:30%:20%:20% at the seedling, flowering and fruiting, green ripening, and color change stages, respectively.

Uniform-sized tomato seedlings with one young fruit were chosen to conduct high-temperature and nitrogen experiments during the flowering and fruiting stage. Three levels of daily minimum/daily maximum temperature were adopted, namely control (CK; 18°C/28°C), sub-high temperature (SHT; 25°C/35°C), and high-temperature (HT; 30°C/40°C) stress, and the duration lasted for 5 days. All seedlings were divided into 15 groups with each group containing 9 pots (**Table 1**). Plants uptaking nitrogen after 5 days were moved into artificial climate chambers (BDW 40, Conviron, Canada). During the treatment, the relative humidity was set at 50-80%, and the light was 6:00-18:00 (daytime) with photosynthetic active radiation between 0 to 1000 μmol m^-2^ s^-1^.

**Table 1:** Treatment combination of nitrogen level and temperature level for tomato in the greenhouse.

Nitrogen treatment (kg hm^-2^)	Temperature treatment (daily maximum/daily minimum)		
	CK (28°C/18°C)	SHT (35°C/25°C)	HT (40°C/30°C)
N_1_:0N (0kg hm^-2^)	CKN_1_	SHTN_1_	HTN_1_
N_2_:0.5N (125kg hm^-2^)	CKN_2_	SHTN_2_	HTN_2_
N_3_:0.75N (187.5kg hm^-2^)	CKN_3_	SHTN_3_	HTN_3_
N_4_:1.0N (250kg hm^-2^)	CKN_4_	SHTN_4_	HTN_4_
N_5_:1.25N (312.5kg hm^-2^)	CKN_5_	SHTN_5_	HTN_5_

CK, SHT and HT are the control, sub-high temperature and high temperature treatment, respectively.

After the treatments, all plants were removed to the Velno-type greenhouse for the recovery period. Measurements were taken with 3 random pots at the end of treatments and conducted on the third to fifth functional leaves. Additionally, to measure the fruit quality, three tomato fruits of relatively uniform size and without mechanical damage were chosen at maturity for each treatment. Water is supplied twice a day during the treatment and at least every two days (in most cases once or twice a day) during the seedling and recovery stages. All plants were irrigated to 80% field capacity (monitoring by soil moisture content tester) to avoid water deficiency.

### 2.2 The methods of measurement

#### 2.2.1 Gas exchange parameters and SPAD value

Gas exchange parameters, including the stomatal conductance (*g*_s_) and intercellular carbon dioxide concentration (*C*_i_), were measured by an LI-6400XT photosynthesis system (LI-COR Inc., Lincoln, NE, USA) from 9:00-11:00. The leaf chamber temperature was set to 25°C, the relative humidity was set to 65%, and the CO_2_ concentration was kept at 390 μmol mol^–1^. Photosynthetically active radiation (PAR) was set to 1800, 1600, 1400, 1200, 1000, 800, 600, 400, 200, 150, 100, 50, and 0 μmol m^-2^ s^-1^, respectively. The stomatal limit (L_s_) value was determined by equation (1).


(1) $$L_{s}=1-\frac{C_{i}}{C_{a}}$$

Where *C*_a_ indicates the atmospheric CO_2_ concentration.

And


(2) $$water–use\;efficiency\;\left(WUE\right)=\frac{net\;photosynthetic\;rate\;\left(P_{n}\right)}{transpiration\;rate\;\left(T_{r}\right)}$$

The *P*_Nmax_ was obtained by the photosynthesis-light response curves based on the photosynthetic electron transport of photosystem II in C_3_ and C_4_ species (Ye, 2012; Ye et al., 2013).

SPAD value was measured by Chlorophyll Meter Model (SPAD-502, Konica Minolta, Japan).

#### 2.2.2 Nitrogen metabolism and nutrients

The third to fifth function leaves were picked from the top of tomato plants, frozen rapidly with liquid nitrogen for 15 min, and then store at -20°C for measurement. Nitrate reductase (NR, EC 1.6.6.1) was determined according to the method of Miflin and Habash (2002). NR can be expressed in terms of the amount of nitroso-nitrogen produced, which has a maximum absorption peak at 540nm. Glutamine synthetase (GS, EC 2.7.7.42) activity was measured using the method of Du et al. (2008). One unit of GS activity was determined by the absorbance values at 540 nm using the spectrophotometer (UV-1800, Shimadzu, Japan). The NR and GS activities were calculated per germ of fresh weight (FW).

Soluble protein was determined by the coomassie brilliant blue method (Sedmak and Grossberg, 1977), which was measured by the absorbance of the solution at 595 nm, and the protein content was found by the standard curve. The content of free amino acids was determined by the ninhydrin chromogenic method according to Friedman (2004), which was colorimetric at 570 nm.

#### 2.2.3 Morphological characters

Plant height, stem diameter, main root length, and leaf area were measured by tape ruler, electronic vernier caliper, rule, and leaf area meter (LI-3100C, Li-Cor, Inc., USA), respectively. Plant height growth, stem diameter height growth, and leaf area index (LAI) were calculated as:


(3) $$Plant\;height\;growth=Plant\;height_{5d}-Plant\;height_{0d}$$

(4) $$Stem\;diameter\;growth=Stem\;diameter_{5d}-Stem\;diameter_{0d}$$

(5) $$LAI=\frac{leaf\;area\;per\;plant\times total\;number\;of\;plants\;per\;unit\;of\;land\;area}{unit\;area\;of\;land}$$

Where 0d and 5d are the value measured at 0d and 5d, respectively.

#### 2.2.4 Fruit quality

Extrinsic quality (single fruit weight, fruit length, maximum and minimum fruit diameter, etc.) and intrinsic quality (Vitamin C, anthocyanin, organic acid content, and soluble sugar content, etc.) are two aspects of fruit quality (Ashalley, 2014). Fruit hardness, single fruit weight, and maximum and minimum fruit diameter were measured by fruit hardness meter (GY-4, Aipli, China), electronic balance (ES-220D, China), and electronic Vernier caliper, respectively. Fruit shape index and yield were calculated as:


(6) $$Fruit\;shape\;index=\frac{fruit\;longitudinal\;diameter}{fruit\;horizontal\;diameter}$$

(7) Yield=plants per hectare×normal fruits per plant×single weight fruit 

The content of Vitamin C (VC), titratable acid, and soluble sugar was determined by the 2,6-dichloroindophenol titrimetric (JAOAC, 1984), micro alkaline titration (Wei, 2020), and the anthrone method (Wei, 2020), respectively. Lycopene was determined according to Zhao et al. (2022). Nitrate (NO_3_^-^) was determined according to Wang et al. (2017). Soluble solids were measured by a hand-held refractometer (ATC, Aipli, China).

### 2.3 Statistical analysis

#### 2.3.1 Variance analysis

All data were the mean ± standard deviation (SD) of 3 biological replications. SPSS 24.0 (SPSS, Chicago, IL, USA) for one–way analysis of variance (ANOVA), interaction analysis, Duncan’s multiple comparisons (at *P*=0.05), correlation analysis, and principal component analysis.

#### 2.3.2 Principal component analysis

The indicators of tomato photosynthesis, nitrogen metabolism, nutrients, growth, and fruit quality were evaluated by correlation analysis and principal component analysis. The comprehensive index (CI) is constructed by the indicators with high contribution rates and most of the information in principal component analysis (Li et al., 2019). The standardized values, weights, and comprehensive index are calculated as follows.

This study uses the opposite difference approach (8) to normalize the indicators so that their ranges were between [0,1].


(8) Xi'={Xi−XminiXmaxi−XminiPositive indicatorsXmaxi−XiXmaxi−XminiNegative indicators

Where Xi' is the standardized value of the i^th^ indicator, (i=1, 2, 3, …, 23); $X_{mini}$ is the minimum value of the i^th^ indicator; $X_{maxi}$ is the maximum value of the i^th^ indicator.

The weight of the indicators in Principal Component A_k_ can be calculated as:


(9) $$W_{ki}=\frac{C_{ki}}{\sqrt{E_{k}}}\;$$

Where $W_{ki}$ is the weight of the i^th^ indicator in A_k_, (k=1, 2, …., p); $C_{ki}$ is the loadings of the i^th^ indicator in A_k_; $E_{k}$ is the eigenvalues of A_k_.

The results of the comprehensive index in Principal Component A_k_ are obtained as:


(10) $$CI_{k}=\sum_{i=1}^{n}W_{ki}\times X_{i}^{'}$$

Where $CI_{k}$ is the comprehensive index of A_k_.

The results of comprehensive evaluation in Principal Component analysis are obtained as:


(11) $$W_{k}=P_{k}/\sum_{k=1}^{n}P_{k}$$

(12) $$CI=\sum_{k=1}^{n}W_{k}\times CI_{k}$$

Where $P_{k}$ is the contribution rate of Principal Component A_k_; $W_{k}$ is the weight of Principal Component A_k_.

## 3 Results

### 3.1 Effects of nitrogen application on gas exchange parameters and SPAD value under high-temperature stress

High-temperature (HT) stress had negative impacts on the gas exchange parameters of tomato leaves, which was characterized by the decrease in *P*_Nmax_, *g*_s_, L_S_, and WUE but the increase in *C*_i_ (**Table 2**). Compared to the CK and HT, the *P*_Nmax_, *g*_s_, L_S_, and WUE in the SHT group increased significantly, but the *C*_i_ was reduced significantly (*P*<0.05). The *P*_Nmax_, *g*_s_, L_S_, *C*_i_, and WUE were significantly influenced by appropriate nitrogen applications under different temperature treatments. Maximum *P*_Nmax_, *g*_s_, L_S_ and WUE occurred at CKN_3_, SHTN_3_, and HTN_2_, respectively, whereas the *C*_i_ was the lowest. *P*_Nmax_, *g*_s_, and WUE in HTN_2_ were lower than those of the CKN_3_. However, those parameters significantly increased by 15.54%, 22.45%, and 13.96%, respectively, under SHTN_3_. L_S_ showed no significant difference among CKN_3_, SHTN_3_, and HTN_2_. Furthermore, *C*_i_ under HTN_2_ was 5.43% higher than that of CKN_3_, but no significant differences between CKN_3_ and SHTN_3_ (*P*>0.05).

**Table 2:** Effects of nitrogen application on gas exchange parameters of tomato leaves under high-temperature stress.

Treatment	*P*_Nmax_ (μmol m^-2^s^-1^)	*g*_s_ (mol m^-2^ s^-1^)	*C*_i_ (μmol mol^-1^)	L_s_	WUE (μmol mmol^-1^)
CKN_1_	6.34 ± 0.07 k	0.04 ± 0.01 i	363.82 ± 1.82 a	0.09 ± 0.01 d	1.11 ± 0.13 hi
CKN_2_	8.62 ± 0.13 h	0.08 ± 0.01 i	358.63 ± 1.19 b	0.10 ± 0.01 d	1.42 ± 0.05 gh
CKN_3_	14.54 ± 0.08 d	0.33 ± 0.05 ef	329.78 ± 2.48 d	0.18 ± 0.03 bc	2.56 ± 0.17 d
CKN_4_	17.82 ± 0.26 b	0.49 ± 0.04 bc	307.01 ± 2.79 g	0.23 ± 0.03 a	3.94 ± 0.11 b
CKN_5_	7.89 ± 0.14 i	0.06 ± 0.01 i	360.83 ± 2.42 ab	0.10 ± 0.03 d	1.49 ± 0.12 g
SHTN_1_	10.30 ± 0.12 g	0.22 ± 0.05 h	335.29 ± 1.86 c	0.16 ± 0.01 c	1.83 ± 0.14 f
SHTN_2_	14.65 ± 0.39 d	0.37 ± 0.04 de	325.71 ± 1.59 e	0.19 ± 0.01 abc	2.68 ± 0.18 d
SHTN_3_	20.59 ± 0.27 a	0.60 ± 0.05 a	307.37 ± 1.14 g	0.23 ± 0.01 a	4.49 ± 0.24 a
SHTN_4_	18.00 ± 0.09 b	0.50 ± 0.02 b	309.26 ± 1.81 g	0.23 ± 0.02 a	3.90 ± 0.03 b
SHTN_5_	16.03 ± 0.17 c	0.43 ± 0.02 cd	317.60 ± 1.45 f	0.21 ± 0.02 ab	3.23 ± 0.09 c
HTN_1_	6.11 ± 0.12 k	0.04 ± 0.01 i	363.08 ± 3.22 a	0.09 ± 0.01 d	1.15 ± 0.15 hi
HTN_2_	14.69 ± 0.20 d	0.38 ± 0.05 de	323.68 ± 2.53 e	0.19 ± 0.02 abc	2.64 ± 0.11 d
HTN_3_	12.74 ± 0.05 e	0.30 ± 0.01 fg	330.49 ± 0.98 d	0.17 ± 0.01 bc	2.26 ± 0.12 e
HTN_4_	10.85 ± 0.12 f	0.24 ± 0.01 gh	332.86 ± 1.28 cd	0.17 ± 0.02 bc	1.92 ± 0.07 f
HTN_5_	7.03 ± 0.11 j	0.05 ± 0.01 i	361.66 ± 1.57 ab	0.10 ± 0.01 d	1.20 ± 0.09 hi
Temperature (T)	**	**	**	**	**
Nitrogen (N)	**	**	**	**	**
T x N	**	**	**	**	**

Different lowercase letters indicate significant differences among treatments at the P< 0.05 level by Duncan’s test. CK, SHT and HT are the control, sub-high temperature and high temperature treatment, respectively. Values are mean ± SD (n = 3). ** indicates significance at P ≤ 0.01.

**Figure 1:**
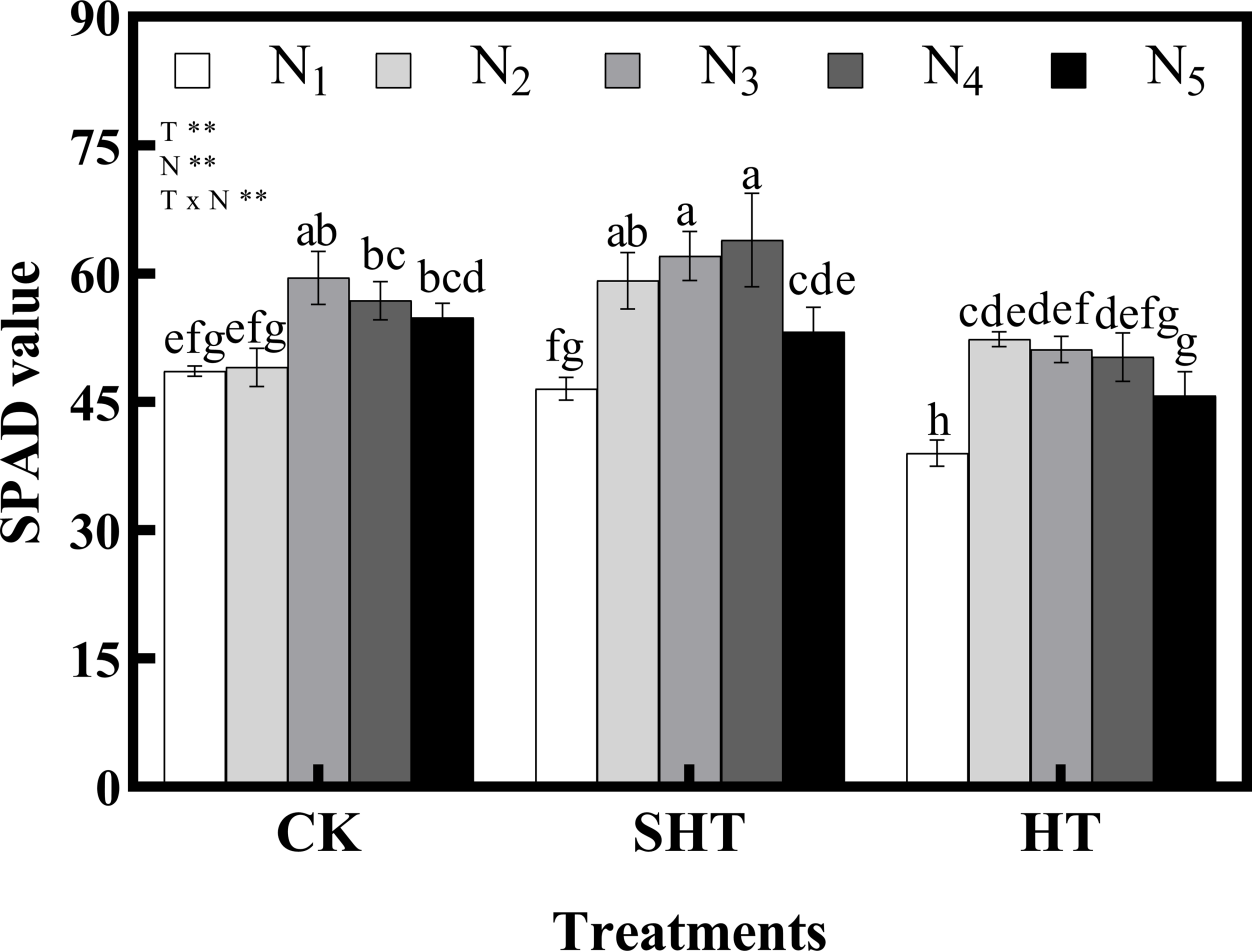
Effects of nitrogen application on SPAD value of tomato leaves under high-temperature stress. Different lowercase letters indicate significant differences among treatments at the *P*< 0.05 level by Duncan’s test. CK, SHT and HT are the control, sub-high temperature and high temperature treatment, respectively. T, temperature; N, nitrogen. Values are mean ± SD (n = 3). ** indicates significance at *P* ≤ 0.01.

The SPAD value was also influenced by HT stress (**Figure 1**). Meanwhile, appropriate nitrogen application significantly mitigated the effects of HT stress on SPAD value, with a marked improvement in CKN_3_, SHTN_4_, and HTN_2_. SPAD value under CKN_3_ was 13.70% higher than that of HTN_2_, while no significant difference with SHTN_3_.

### 3.2 Response of nitrogen application on nitrogen metabolism and nutrients to high-temperature stress

**Figure 2:**
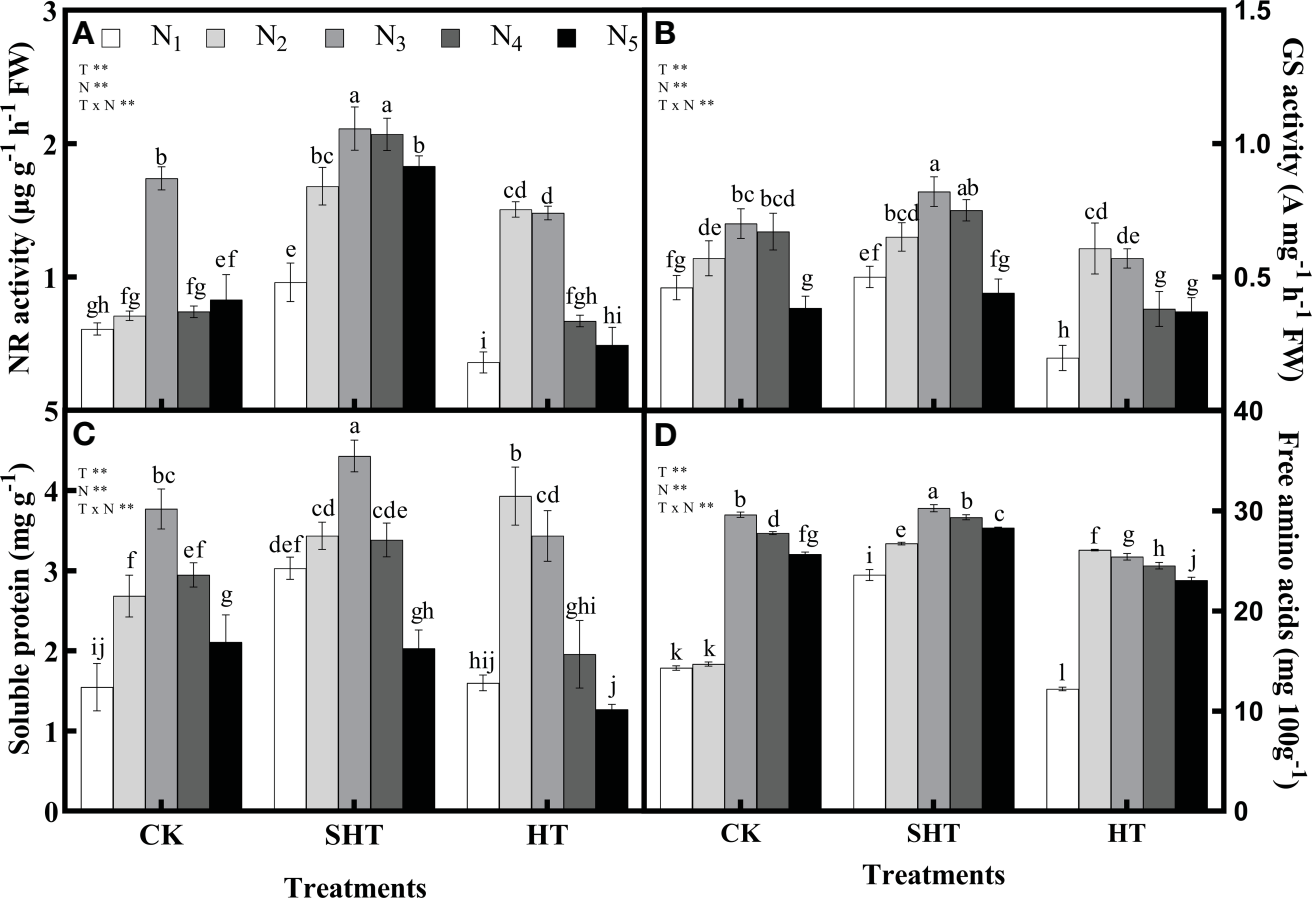
Effects of nitrogen application on the nitrate reductase (NR) activity **(A)**, glutamine synthetase (GS) activity **(B)**, soluble protein **(C)**, and free amino acids **(D)** in tomato leaves under high-temperature stress. Different lowercase letters indicate significant differences among treatments at the *P*< 0.05 level by Duncan’s test. CK, SHT and HT are the control, sub-high temperature and high temperature treatment, respectively. T, temperature; N, nitrogen. Values are mean ± SD (n = 3). ** indicates significance at *P* ≤ 0.01.

The responses of nitrogen application on nitrogen metabolism and nutrients to HT stress were shown in **Figure 2**. The NR, GS, soluble protein, and free amino acids in CK were significantly greater than in HT but were lower than in SHT, without nitrogen application. Appropriate nitrogen application significantly increased the contents of NR, GS, soluble protein, and free amino acids in the leaves of tomato plants. The NR, GS, soluble protein and free amino acids were highest in CKN_3_, SHTN_3_, and HTN_2_, respectively. Compared to CKN_3_, the activity of NR and GS was greater than HTN_2_, whereas was significantly reduced by 21.26% and 17.14%, respectively, under SHTN_3_. Similar to NR and GS, free amino acids decreased significantly by 11.91% in HTN_2_ but improved significantly by 2.19% in SHTN_3_. Soluble protein under CKN_3_ was 14.90% lower than that of SHTN_3_. However, for soluble protein, there was no significant difference between CKN_3_ and HTN_2_ treatments.

### 3.3 Effects of nitrogen application on morphological characters under high-temperature stress

HT stress was reflected in the morphological characters (**Table 3**). Without nitrogen application, compared to CK, the plant height growth, stem diameter growth, main root length, and LAI were inhibited under HT, whereas were improved under SHT. Nitrogen application significantly affected the morphological characters of tomato plants under high-temperature stress. The plant height growth in CK increased with the increase of N_1_-N_3_, while they decreased with a further increase of N_4_-N_5_. Maximum plant height growth occurred at CKN_3_, SHTN_4_, and HTN_3_, respectively. Similarly, stem diameter growth was the highest in CKN_3_, SHTN_3_, and HTN_3_. The stem diameter growth in the CKN_3_ was greater than that of the HTN_3_, while significantly decreasing by 57.89% under the SHTN_3_. The main root length and LAI also had appropriate nitrogen application under different temperature treatments. Compared to CK, the maximum main root length and LAI were significantly reduced by 6.76% and 20.29%, respectively, under HT conditions. In contrast, these two indicators under SHT were significantly increased by 3.72% and 50.72%, respectively, compared to the CK group.

**Table 3:** Effects of nitrogen application on morphological characters of tomato leaves under high-temperature stress.

Treatment	Plant height growth (cm)	Stem diameter growth (mm)	Main root length (cm)	LAI
CKN_1_	3.04 ± 0.07 hi	-0.27 ± 0.13 e	5.67 ± 0.10 g	0.89 ± 0.06 g
CKN_2_	3.29 ± 0.02 h	0.34 ± 0.08 bcd	5.98 ± 0.05 f	1.04 ± 0.06 f
CKN_3_	4.45 ± 0.18 e	0.57 ± 0.02 b	8.78 ± 0.05 b	1.22 ± 0.06 e
CKN_4_	3.71 ± 0.08 g	0.50 ± 0.07 bc	8.88 ± 0.14 b	1.38 ± 0.06 d
CKN_5_	4.15 ± 0.34 f	-0.16 ± 0.27 e	5.09 ± 0.09 hi	1.14 ± 0.06 ef
SHTN_1_	5.33 ± 0.12 d	-0.08 ± 0.20 e	6.26 ± 0.05 e	1.45 ± 0.04 d
SHTN_2_	5.91 ± 0.06 c	0.25 ± 0.08 d	6.45 ± 0.02 e	1.72 ± 0.02 c
SHTN_3_	6.30 ± 0.10 b	0.90 ± 0.03 a	5.20 ± 0.15 hi	1.92 ± 0.04 b
SHTN_4_	7.49 ± 0.14 a	0.54 ± 0.02 bc	9.21 ± 0.26 a	2.08 ± 0.07 a
SHTN_5_	6.22 ± 0.02 b	0.31 ± 0.10 bcd	5.36 ± 0.04 h	1.62 ± 0.06 c
HTN_1_	2.55 ± 0.09 j	-0.50 ± 0.04 f	5.08 ± 0.10 hi	0.75 ± 0.05 h
HTN_2_	2.84 ± 0.17 ij	0.31 ± 0.02 cd	8.28 ± 0.28 c	0.84 ± 0.03 gh
HTN_3_	3.00 ± 0.11 i	0.34 ± 0.05 bcd	6.73 ± 0.05 d	1.10 ± 0.07 f
HTN_4_	2.76 ± 0.10 ij	-1.07 ± 0.09 h	6.18 ± 0.11 ef	0.81 ± 0.06 gh
HTN_5_	2.27 ± 0.14 k	-0.80 ± 0.09 g	4.97 ± 0.10 i	0.73 ± 0.04 h
Temperature (T)	**	**	**	**
Nitrogen (N)	**	**	**	**
T x N	**	**	**	**

Different lowercase letters indicate significant differences among treatments at the P< 0.05 level by Duncan’s test. CK, SHT and HT are the control, sub-high temperature and high temperature treatment, respectively. Values are mean ± SD (n = 3). LAI, leaf area index. ** indicates significance at P ≤ 0.01.

### 3.4 Response of nitrogen application on fruit quality to high-temperature stress

The effects of nitrogen application on fruit shape index, fruit hardness, and yield of tomatoes under HT stress were shown in **Table 4**. The fruit shape index showed no significant difference among treatments of N_2_-N_3_ except N_4_-N_5_. However, fruit hardness was no significant difference among different temperature treatments. The yield in CK was significantly greater than in HT but was no significant difference in SHT, without nitrogen application. Appropriate nitrogen application could enhance high-temperature stress tolerance. The yield was the highest in N_4_ under CK and SHT treatment, while the highest occurred at N_2_ under HT treatment. Compared to HTN_2_, the yield was significantly increased by 104.54% and 110.04%, respectively, for CKN_4_ and SHTN_4_ treatments.

**Table 4:** Effects of nitrogen application on external quality of tomato fruit under high-temperature stress.

Treatment	Fruit shape index	Fruit hardness (kg cm^-2^)	Yield (t hm^-2^)
CKN_1_	0.96 ± 0.07 abc	2.16 ± 0.20 abcd	4.54 ± 0.89 ef
CKN_2_	0.93 ± 0.04 abcde	2.12 ± 0.38 abcd	9.47 ± 2.13 c
CKN_3_	0.89 ± 0.03 bcdef	1.45 ± 0.05 cd	15.38 ± 2.67 b
CKN_4_	0.92 ± 0.01 abcde	1.51 ± 0.08 cd	18.94 ± 4.32 ab
CKN_5_	0.94 ± 0.02 abcd	1.33 ± 0.24 d	7.45 ± 1.31 cde
SHTN_1_	0.97 ± 0.01 ab	2.53 ± 0.12 ab	5.01 ± 0.22 def
SHTN_2_	0.83 ± 0.02 def	3.03 ± 0.22 a	9.35 ± 0.93 c
SHTN_3_	0.84 ± 0.07 cdef	2.67 ± 0.31 ab	15.76 ± 2.55ab
SHTN_4_	0.91 ± 0.05 abcde	1.82 ± 0.22 bcd	19.45 ± 1.36 a
SHTN_5_	0.96 ± 0.03 abc	2.55 ± 0.63 ab	4.21 ± 1.05 efg
HTN_1_	1.03 ± 0.06 a	2.68 ± 0.07 ab	0.66 ± 0.02 g
HTN_2_	0.86 ± 0.10 bcdef	2.60 ± 0.32 ab	9.26 ± 1.06 c
HTN_3_	0.83 ± 0.04 cdef	2.51 ± 0.72 ab	8.68 ± 0.29 cd
HTN_4_	0.80 ± 0.07 ef	2.42 ± 0.03 abc	7.73 ± 0.36 cde
HTN_5_	0.76 ± 0.10 f	2.85 ± 1.11 a	2.27 ± 0.97 fg
Temperature (T)	*	**	**
Nitrogen (N)	**	n.s.	**
T x N	*	n.s.	**

Different lowercase letters indicate significant differences among treatments at the P< 0.05 level by Duncan’s test. CK, SHT and HT are the control, sub-high temperature and high temperature treatment, respectively. Values are mean ± SD (n = 3). n.s., *, ** indicate non-significance and significance at P ≤ 0.05 and 0.01, respectively.

**Figure 3:**
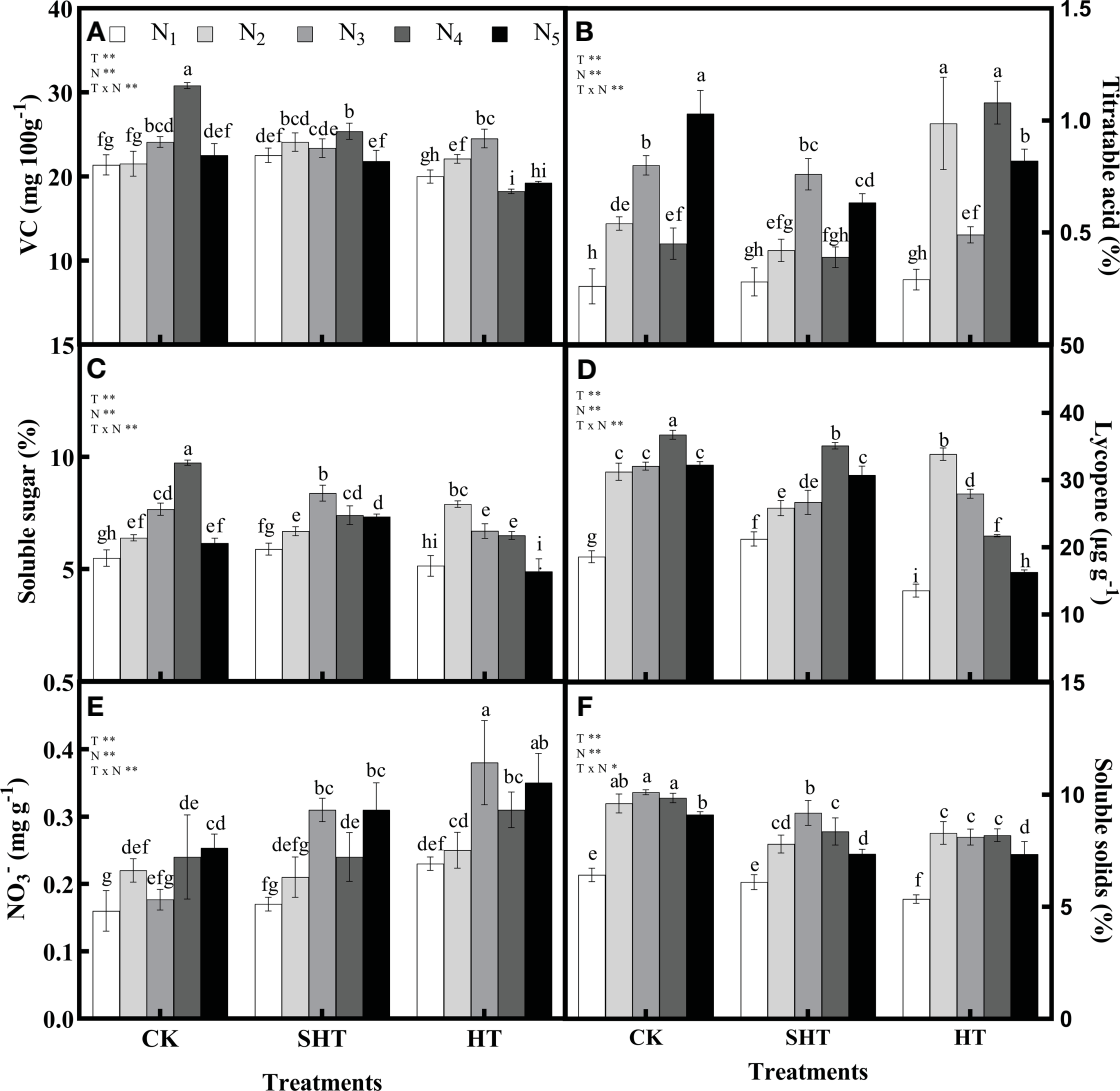
Effects of nitrogen application on the Vitamin C (VC; A), titratable acid (B), soluble sugar (C), lycopene (D), nitrate (NO_3_^-^; E), and soluble solids (F) of tomato fruit under high-temperature stress. Different lowercase letters indicate significant differences among treatments at the P < 0.05 level by Duncan's test. CK, SHT and HT are the control, sub-high temperature and high temperature treatment, respectively. T, temperature. N, nitrogen. Values are mean ± SD (n = 3). ** indicates significance at P ≤ 0.01.

HT stress inhibited the intrinsic quality of tomato fruit, while nitrogen application could significantly reduce the negative effects of HT stress (**Figure 3**). The VC, soluble sugar, lycopene, and soluble solids in CK and SHT increased with the increase of N_1_-N_4_, while they decreased with a further increase of N_5_. However, these four indicators in HT increased with the increase of N_1_-N_3_, while they decreased with a further increase of N_4_-N_5_. Maximum VC occurred at N_4_, N_4_, and N_3_, respectively, for CK, SHT, and HT conditions. Compared to CKN_4_, the VC was significantly decreased by 17.68% and 20.47%, respectively, for SHTN_4_ and HTN_3_ treatments. Similarly, the contents of soluble sugar, lycopene, and soluble solids in the SHT and HT groups were lower than those of the CK group. On the contrary, the titratable acid and NO_3_^-^ were lowest in N_3_/N_4_, N_3_/N_4_, and N_2_/N_3_, respectively, for CK, SHT, and HT conditions. However, minimum titratable acid and NO_3_^-^ were no significant differences among treatments.

### 3.5 Comprehensive evaluation of tomato yield, fruit quality, and related traits under different treatments

#### 3.5.1 Correlation analysis between different traits

**Figure 4:**
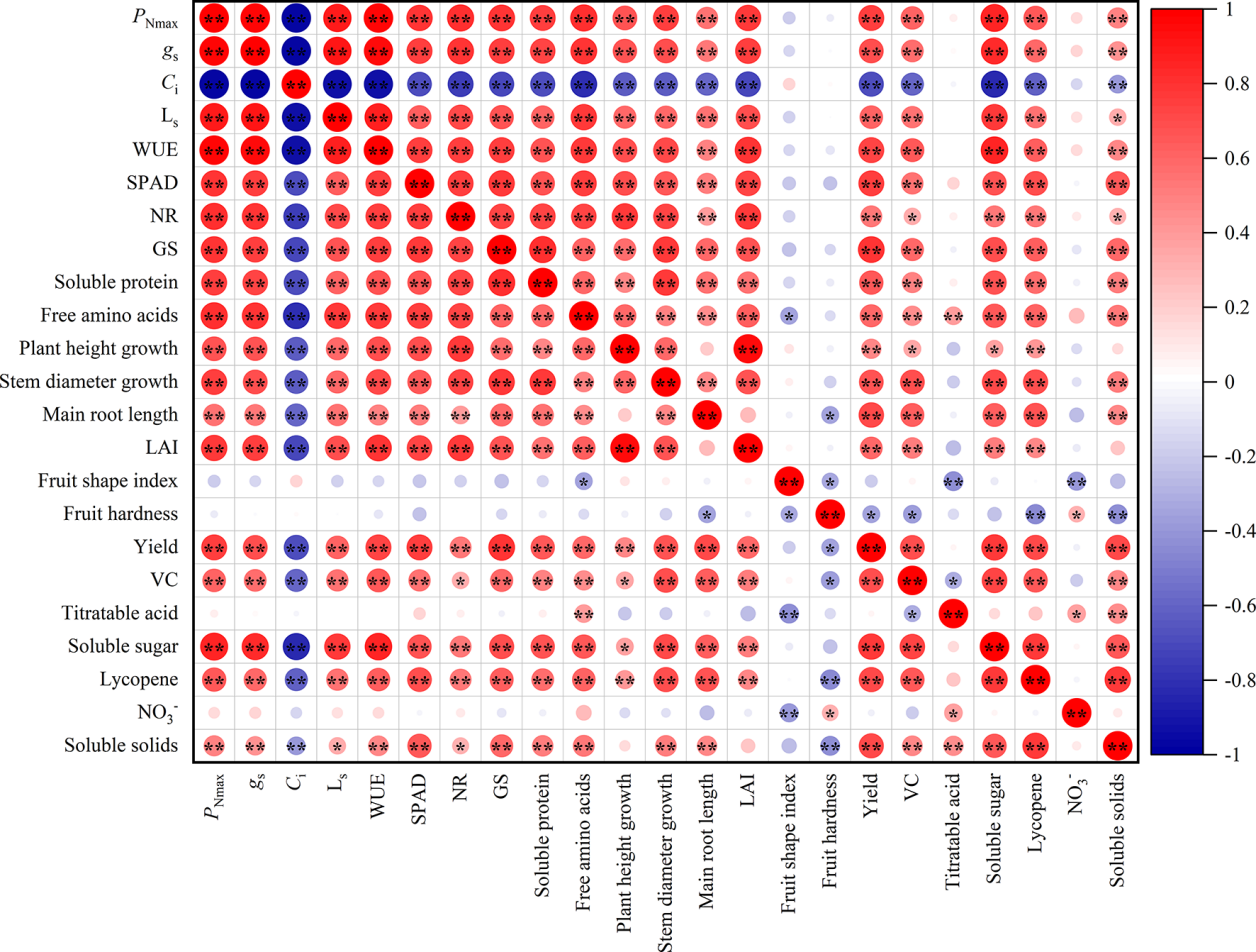
Correlation analysis among leaves and fruit indexes of tomato (n = 45). *P*_Nmax_, the maximum net photosynthetic rate. *g*_s_, stomatal conductance. *C*_i_, carbon dioxide concentration. L_S_, stomatal limit value. WUE, water-use efficiency. NR, nitrate reductase. GS, glutamine synthetase. LAI, leaf area index. VC, Vitamin C; NO_3_^-^, nitrate. * and ** indicate the statistical significance exceeding 95% and 99%, respectively.

**Figure 4** showed that there were different degrees of positive and negative correlations between the trait indicators of the different treatments. The absolute values of the correlation coefficients for most indicators ranged from 0.60 to 0.98. Gas exchange parameters and SPAD value were highly significantly correlated (*P*<0.01), with the absolute values of the correlation coefficients ranging from 0.60 to 0.98. It can be assumed that the gas exchange parameters and SPAD value provide 60% to 98% of the common information. Similarly, indicators of nitrogen metabolism and nutrients had a highly significant positive correlation, providing 61% to 81% of the common information. The majority of morphological characters showed highly significant positive correlations, with correlation coefficients ranging from 0.45 to 0.59. Furthermore, the correlation coefficients for yield, VC, soluble sugar, soluble solids, and lycopene among the fruit quality varied from 0.47 to 0.77, giving 47% to 77% of the common information.

The 23 indicators measured contained a great deal of information and were highly correlated with each other. However, they were not suitable for determining indicators of nitrogen regulation under HT stress. Therefore, a principal component analysis was introduced to reduce the number of indicators to a few aggregated indicators for further analysis, without losing too much information.

#### 3.5.2 Principal component and fitting analysis

The principal component analysis of the 23 indicators showed that the cumulative variance contribution of the first four components had reached 86.255%, satisfying the principle of eigenvalues greater than 1 and cumulative contribution greater than 85% (**Table 5**). **Table 5** showed that the first principal component could characterize 58.676% of the information, with *P*_Nmax_, WUE, *g*_s_, *C*_i_, L_s_, SPAD value, GS, soluble sugar, yield, stem diameter growth, NR, free amino acids, soluble protein, LAI, and lycopene having larger absolute values of the eigenvectors. *P*_Nmax_ is the strongest indicator of photosynthesis and has the largest eigenvector, containing information common with *g*_s_, WUE, *C*_i_, L_s_, and SPAD value. Thus, *P*_Nmax_ was selected. GS can represent NR, free amino acids, and soluble protein as an indicator of the strength of nitrogen metabolism and be therefore selected. Similarly, yield, stem diameter growth, soluble sugar, and lycopene were selected. The larger absolute values of eigenvectors in the second principal components were fruit shape index, NO_3_^-^ and titratable acid. However, the third and fourth principal components were fruit hardness and soluble solids, plant height growth and main root length, respectively.

**Table 5:** Contribution rate of principle components and eigenvectors of each index.

Principle component factor		PC1	PC2	PC3	PC4
Eigenvalue		13.495	2.761	2.494	1.088
Variance contribution rate (%)		58.676	12.003	10.846	4.731
Cumulative variance contribution (%)		58.676	70.678	81.524	86.255
Eigenvector	*P*_Nmax_	0.262	-0.094	-0.090	-0.101
	WUE	0.255	-0.063	-0.104	-0.102
	*g*_s_	0.255	-0.111	-0.126	-0.150
	*C*_i_	-0.250	0.100	0.110	0.222
	L_s_	0.249	-0.109	-0.111	-0.190
	SPAD value	0.247	-0.015	0.055	0.298
	GS	0.245	0.038	0.007	0.019
	Soluble sugar	0.241	0.036	0.137	-0.253
	Yield	0.241	0.114	0.172	-0.039
	Stem diameter growth	0.225	0.165	-0.066	0.051
	NR	0.225	-0.117	-0.188	0.250
	Free amino acids	0.224	-0.221	0.049	0.142
	Soluble protein	0.223	-0.019	-0.019	-0.031
	LAI	0.214	0.060	-0.300	0.279
	Lycopene	0.214	0.119	0.257	0.099
	VC	0.200	0.262	0.067	-0.286
	Plant height growth	0.188	0.066	-0.331	0.421
	Main root length	0.179	0.192	0.195	-0.312
	NO_3_^-^	-0.011	0.484	-0.046	0.152
	Fruit shape index	0.055	-0.476	0.175	-0.051
	Soluble solids	0.175	-0.013	0.430	0.160
	Fruit hardness	0.073	0.343	0.403	0.238
	Titratable acid	-0.018	0.374	-0.399	-0.281

P_Nmax_, the maximum net photosynthetic rate; g_s_, stomatal conductance; C_i_, carbon dioxide concentration; L_S_, stomatal limit value; WUE, water-use efficiency; NR, nitrate reductase; GS, glutamine synthetase; LAI, leaf area index; VC, Vitamin C; NO_3_^-^, nitrate.

Although the four principal components above combined most of the indicator information, the characteristic information overlapped to some extent, and the number of input parameters was large. Therefore, further screening was needed. *P*_Nmax_, yield, soluble sugar, GS, stem diameter growth, and lycopene were selected to construct the CI, considering the correlation, the contribution of principal component analysis and eigenvectors, the biological significance of the indicators and the ease of determination.

Soluble sugar, GS, yield, *P*_Nmax_, stem diameter growth, and lycopene were again subjected to principal component analysis and the eigenvectors obtained were 0.419, 0.417, 0.416, 0.413, 0.400, and 0.384, respectively (**Table 6**). The standardized indicators and eigenvectors were used to CI under different treatments.

**Table 6:** Contribution of principal components and eigenvectors of each index after the secondary screening.

Principle component factor		PC1
Eigenvalue		4.850
Variance contribution rate (%)		80.829
Cumulative variance contribution (%)		80.829
Eigenvector	Soluble sugar	0.419
	GS	0.417
	Yield	0.416
	*P*_Nmax_	0.413
	Stem diameter growth	0.400
	Lycopene	0.384

GS, glutamine synthetase; P_Nmax_, the maximum net photosynthetic rate.


(13) $$CI=0.419X_{soluble\;sugar}^{'}+0.417X_{GS}^{'}+0.416X_{yield}^{'}+0.413X_{P_{Nmax}}^{'}+0.400X_{stem\;diameter\;growth}^{'}+0.384X_{lycopene}^{'}$$

**Figure 5:**
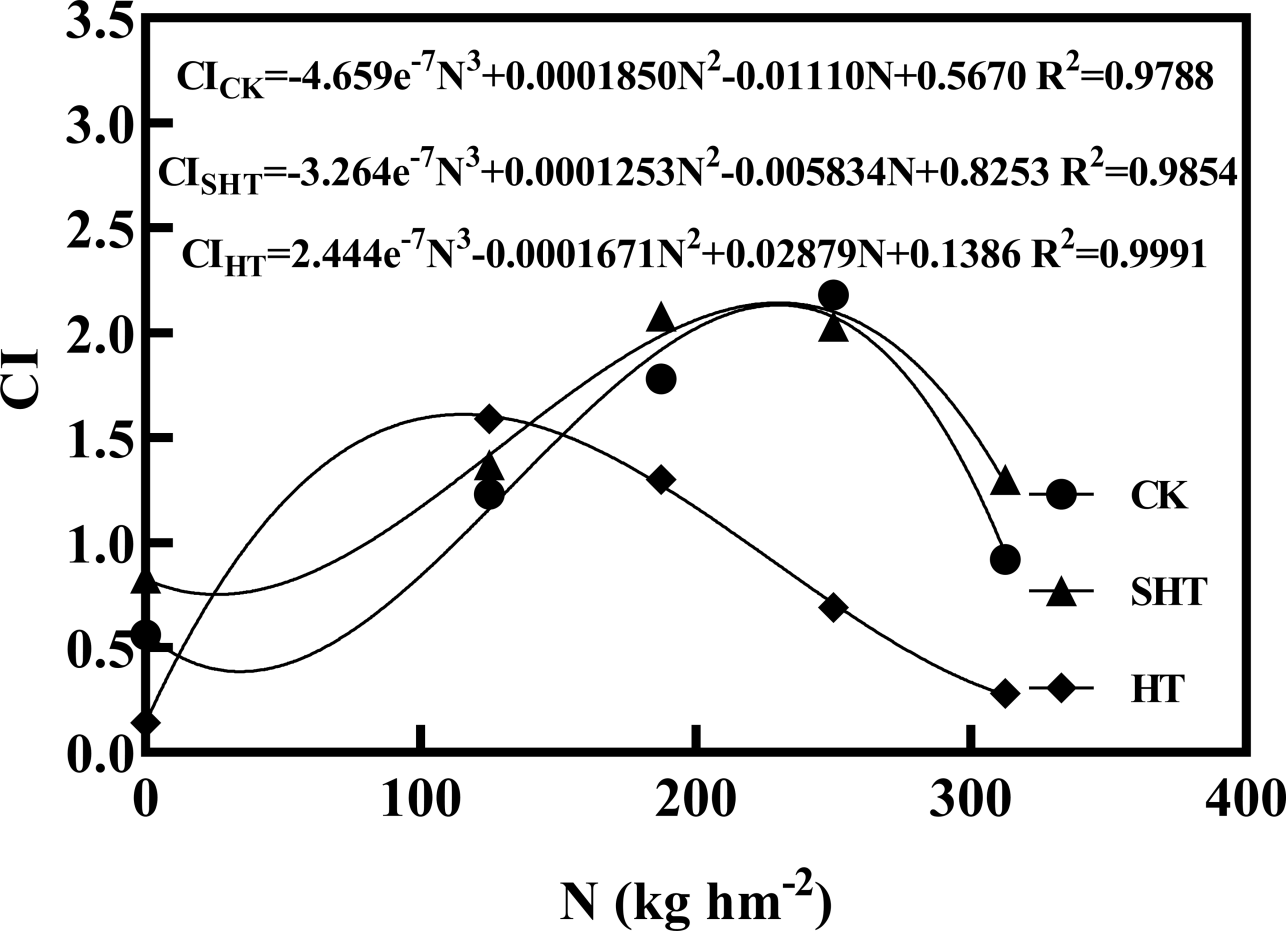
Comprehensive value (CI) of tomatoes as affected by nitrogen application under high-temperature stress. CK, SHT and HT are the control, sub-high temperature and high temperature treatment, respectively. N, nitrogen. R^2^, coefficient of determination.


The CI was highest at moderate nitrogen application under different temperatures, decreasing at low and high nitrogen applications (**Figure 5**). The response fits the regression models:

(14) $$CI_{CK}=\;-4.659e^{-7}N^{3}+0.0001850N^{2}-0.01110N+0.5670\;R^{2}=0.9788\;$$

(15) $$CI_{SHT}=\;-3.264e^{-7}N^{3}+0.0001253N^{2}-0.005834N+0.8253\;R^{2}=0.9854$$

(16) $$CI_{HT}=\;2.444e^{-7}N^{3}-0.0001671N^{2}+0.02879N+0.1386\;R^{2}=0.9991$$

Based on the regression equations, the highest CI of CK, SHT, and HT were obtained at the nitrogen of 230.23 kg hm^2^ (N_3_-N_4_), 230.02 kg hm^2^ (N_3_-N_4_), and 115.32 kg hm^2^ (N_2_), respectively.

## 4 Discussion

High temperature is one of the main disasters affecting the growth, yield, and fruit quality of plants (Mathur et al., 2014). The effect of high-temperature stress on plants receives more attention as the frequency and intensity of high-temperature stress increase globally (IPCC, 2021). Nitrogen plays an important role in plant growth, development, and yield. Appropriate nitrogen application can significantly improve high-temperature stress tolerance (James et al., 2018). In this study, we explored how nitrogen affects tomato plants under high-temperature stress.

First, short-term high-temperature stress affects the photosynthetic process of tomato leaves. Photosynthesis is one of the most fundamental metabolic activities in plants (Yin et al., 2006). The *P*_Nmax_ was inhibited under high-temperature (HT) stress, accompanied by different declines of *g*_s_, L_s_, and WUE (**Table 2**). However, *C*_i_ increased, which indicated that the decrease was due to non-stomatal limitation and mainly resulted from photosystem damage, and inhibition of ribulose bisphosphate (Rubisco) (Farquhar and Sharkey, 1982; Liu et al., 2013). In addition, HT also significantly decreased the SPAD value of tomato leaves (**Figure 2**), which reflected that the chloroplast development and photosynthetic performance were inhibited (Lu et al., 2019). Interestingly, short-term sub-high temperature (SHT) stress had positive impacts on the photosynthetic process, which was characterized by higher *P*_Nmax_, *g*_s_, L_s_, WUE, SPAD value, and lower *C*_i_, compared to CK. The SHT may improve chloroplast and cytoplasmic structure. Consequently, the Rubisco carboxylation activities and ribulose diphosphate (RuBP) carboxylation regeneration ability increased, thereby maintaining higher photosynthetic efficiency (Dias and Brüggemann, 2010; Han et al., 2019; Li et al., 2020). Nitrogen has positive impacts on photosynthetic reactions (Hamner, 1936; Liu et al., 2013; Ru et al., 2022). Appropriate nitrogen application not only can alleviate the damage of Chl-containing structure and function by high-temperature stress but also maintain the photosynthetic capacity of chloroplasts under high-temperature stress. In our study, maximum *P*_Nmax_, *g*_s_, L_s_, WUE, and SPAD value occurred at N_2_ under HT treatments. Similarly, these photosynthetic parameters were the highest in N_3_, for CK and SHT stress. High-temperature stress was the main cause of the decrease in photosynthetic rate when nitrogen was overused, which was similar to the result of Han et al. (2019).

Second, nitrogen metabolism is tightly associated with nitrogen uptake and photosynthesis and is also closely related to the ultimate life state of the plants, such as growth and development under stress conditions (Xu and Zhou, 2006; Liu et al., 2013; Shu et al., 2016). In this study, short-term HT stress significantly hampered nitrogen metabolism, which further influenced the content of nutrients (**Figure 2**). Previous studies suggested that the decrease in nitrogen metabolism was due to the increase of NH_4_^+^ accumulation in plants, which hindered ammonia assimilation and ATP synthesis required for nitrate (Crawford and Glass, 1998; Yuan et al., 2017). However, under short-term SHT stress, the content of soluble protein and free amino acids increased in tomato leaves, accompanied by different improvements in the activity of NR and GS. These results showed that short-term SHT stress could stimulate enzyme activity in plants. Appropriate nitrogen can effectively enhance the high-temperature tolerance of tomato plants. The findings had also been confirmed on wheat (Ru et al., 2022), cotton (Iqbal et al., 2020), etc. In this study, NR, GS, soluble protein, and free amino acids were the highest in N_3_-N_4_, N_3_-N_4_, and N_2_-N_3_, respectively, for CK, SHT, and HT stress.

Third, plant morphology is also significantly affected by high-temperature stress (Źróbek-Sokolnik, 2012; Fahad et al., 2016). The plant height growth, stem diameter growth, main root length, and LAI were remarkably declined in tomatoes (**Table 3**), which were recognized as sensitive to HT stress. In contrast, short-term SHT stress improved plant morphology. An early study indicated that the maintenance of normal transfer and distribution of photosynthetic products in tomatoes ensured plant growth and development (Xu et al., 2020). Plant morphology changed with nitrogen levels (Boussadia et al., 2010; Bhuvaneswari et al., 2014; Razaq et al., 2017). For example, the results of LAI were consistent with those of previous studies, which found that moderate nitrogen with increased LAI (Meier and Leuschner, 2008). In addition, appropriate nitrogen can effectively alleviate the symptoms brought by high-temperature stress. The high LAI of appropriate nitrogen under high-temperature stress was caused by the increase of the photosynthetic rate during growth and the increase of facilitating nutrient uptake.

Therefore, tomato plants are affected by many factors. The fruit quality is also sensitive to changes in the environment (Gajc-Wolska et al., 2008; Alenazi et al., 2020). In our study, under HT stress, the yield and intrinsic fruit quality were both significantly decreased (**Table 4**; **Figure 3**). This result was also consistent with Hernández et al. (2022). Although the intrinsic quality was also obviously reduced after SHT stress, which was characterized by lower VC, soluble sugar, lycopene, and soluble solids, SHT stress had an improvement in yield. These results indicated that short-term SHT stress maintained higher photosynthetic efficiency and nitrogen metabolism to uptake nutrients, thereby maintaining the high growth and yield of tomatoes. However, high yield is not always conducive to good fruit quality (Sansavini and Corelli-Grappadelli, 1996). Appropriate nitrogen can effectively enhance the heat stress tolerance of plants (Iqbal et al., 2020). On one hand, moderate nitrogen can increase the absorbance of light energy by increasing the leaf area, thus increasing the rate of photosynthesis (Fois et al., 2009; Gautam et al., 2021). On the other hand, it can enhance the activities of nitrogen metabolism via higher photosynthesis, causing enough nutrients, which in turn increases the yield and fruit quality (Nava et al., 2007; Ru et al., 2022). Consequently, the highest yield and fruit quality occurred at N_3_-N_4_, N_3_-N_4_, and N_2_-N_3_, respectively, for CK, SHT, and HT stress.

Temperature and nitrogen interaction affects fruit development and quality through the regulation of various plant physiological processes. For example, temperature and nitrogen interaction might regulate the balance between vegetative and reproductive growth in plants. Additionally, the interaction between nitrogen and temperature might influence the activity of enzymes involved in the biosynthesis of plant hormones such as auxins and cytokinins, which play a critical role in fruit development and quality (Ding et al., 2013). The production and signaling of reactive oxygen species (ROS) in plants have been shown to influence fruit development and quality (Tian et al., 2013). Therefore, temperature and nitrogen interaction might influence fruit development and quality via ROS production and signaling. In summary, the mechanism of nitrogen and temperature interaction in co-regulating fruit development and quality involves the regulation of several physiological processes such as plant metabolic rate, gene expression, and hormone signaling, which collectively influence fruit growth, development, and quality.

Principal component analysis is a very useful method for evaluating objects that are influenced by many factors (Wold et al., 1987). Previous studies had demonstrated that using this method to make comprehensive evaluations was feasible (Shi et al., 2021; Xu et al., 2021). According to our results, *P*_Nmax_, yield, soluble sugar, GS, stem diameter growth, and lycopene were selected to construct the CI (**Table 6**). *P*_Nmax_ plays a vital role in photosynthesis (Xu et al., 2013). Meanwhile, GS is tightly associated with some fundamental processes, including nitrogen uptake and photosynthesis (Thum et al., 2003). Soluble sugar is considered an important factor in the fruit quality and anti-adversity of plants, and it is mainly accumulated by photosynthesis (Wahid et al., 2007). Additionally, lycopene also can reflect the intrinsic fruit quality due to the pigment principally responsible for the characteristic deep-red color of ripe tomato fruits, and it also acts as a natural antioxidant (Shi and Maguer, 2000). Therefore, these six indicators could be used to construct the CI to respond to the effects of nitrogen application under high-temperature stress. Based on the CI, the tomato growth, yield, and fruit quality were the highest in 230.23 kg hm^2^ (N_3_-N_4_), 230.02 kg hm^2^ (N_3_-N_4_), and 115.32 kg hm^2^ (N_2_), respectively, at CK, SHT, and HT stress, which can provide scientific contributions for higher yield and better fruit quality of tomato plants in the greenhouse.

## 5 Conclusions

In this study, the growth, yield, and fruit quality of tomato plants were inhibited by short-term high-temperature (HT) stress. However, sub-high temperature (SHT) stress improved growth and yield whereas fruit quality was reduced. Appropriate nitrogen application could enhance the HT stress tolerance of tomato plants. The *P*_Nmax_, *g*_s_, L_S_, WUE, NR, GS, soluble protein, and free amino acids were the highest in CKN_3_, SHTN_3_, and HTN_2_, respectively, whereas *C*_i_, was the lowest. Maximum SPAD value, plant height growth, stem diameter growth, main root length, LAI, yield, VC, soluble sugar, lycopene, and soluble solids occurred at N_3_-N_4_, N_3_-N_4_, and N_2_-N_3_, respectively, for CK, SHT, and HT. The appropriate nitrogen maintained higher photosynthetic efficiency, nitrogen efficiency, and protein synthesis, thereby maintaining the high yield and quality of tomato plants. This study suggested that the optimum nitrogen application for tomato growth, yield, and fruit quality was 230.23 kg hm^2^ (N_3_-N_4_), 230.02 kg hm^2^ (N_3_-N_4_), and 115.32 kg hm^2^ (N_2_), respectively, at CK, SHT, and HT stress.

## Data availability statement

The original contributions presented in the study are included in the article/**Supplementary Material**. Further inquiries can be directed to the corresponding author.

## Author contributions

JL: Methodology, Data curation, Software, Formal analysis, Writing-original draft. ZY: Conceptualization, Methodology, Validation, Supervision, Writing-original draft. FZ: Methodology, Supervision, Writing-review and editing. CL: Supervision, Writing-review and editing. All authors contributed to the article and approved the submitted version.
